# Risk of intracranial meningioma in patients with acromegaly: a systematic review

**DOI:** 10.3389/fendo.2024.1407615

**Published:** 2024-06-11

**Authors:** Amy X. Guo, Asha Job, Donato Pacione, Nidhi Agrawal

**Affiliations:** ^1^ NYU Grossman School of Medicine, NYU Langone Health, New York, NY, United States; ^2^ Division of Neurosurgery, NYU Langone Health, New York, NY, United States; ^3^ Division of Endocrinology, Diabetes and Metabolism, NYU Langone Medical Center/Bellevue Hospital Center New York, New York, NY, United States

**Keywords:** GH, acromegaly, acromegaly and cancer, meningioma, IGF - I

## Abstract

Acromegaly is a rare endocrine disorder caused by hypersecretion of growth hormone (GH) from a pituitary adenoma. Elevated GH levels stimulate excess production of insulin-like growth factor 1 (IGF-1) which leads to the insidious onset of clinical manifestations. The most common primary central nervous system (CNS) tumors, meningiomas originate from the arachnoid layer of the meninges and are typically benign and slow-growing. Meningiomas are over twice as common in women as in men, with age-adjusted incidence (per 100,000 individuals) of 10.66 and 4.75, respectively. Several reports describe co-occurrence of meningiomas and acromegaly. We aimed to determine whether patients with acromegaly are at elevated risk for meningioma. Investigation of the literature showed that co-occurrence of a pituitary adenoma and a meningioma is a rare phenomenon, and the majority of cases involve GH-secreting adenomas. To the best of our knowledge, a systematic review examining the association between meningiomas and elevated GH levels (due to GH-secreting adenomas in acromegaly or exposure to exogenous GH) has never been conducted. The nature of the observed coexistence between acromegaly and meningioma -whether it reflects causation or mere co-association -is unclear, as is the pathophysiologic etiology.

**Systematic review registration:**

https://www.crd.york.ac.uk/prospero/, identifier CRD42022376998.

## Introduction

Acromegaly is a rare endocrine disorder caused by hypersecretion of growth hormone (GH) from a pituitary adenoma. Elevated GH levels stimulate excess production of insulin-like growth factor 1 (IGF-1) which leads to the insidious onset of clinical manifestations (1).

The most common primary central nervous system (CNS) tumors, meningiomas originate from the arachnoid layer of the meninges and are typically benign and slow-growing. Meningiomas are over twice as common in women as in men, with age-adjusted incidence (per 100,000 individuals) of 10.66 and 4.75, respectively (2).

Several reports describe co-occurrence of meningiomas and acromegaly (3). We aimed to determine whether patients with acromegaly are at an elevated risk for meningioma. Investigation of the literature showed that co-occurrence of a pituitary adenoma and a meningioma is a rare phenomenon, and the majority of cases involve GH-secreting adenomas. To the best of our knowledge, a systematic review examining the association between meningiomas and elevated GH levels (due to GH-secreting adenomas in acromegaly or exposure to exogenous GH) has never been conducted.

The nature of the observed coexistence between acromegaly and meningioma – whether it reflects causation or mere co-association – is unclear, as is the pathophysiologic etiology. Meningiomas express both GH and IGF-1 receptors as indicated by *in vitro* and *in vivo* studies in mice (4). These receptors could potentially be therapeutic targets for GH antagonists such as pegvisomant (5, 6). In addition, somatostatin receptors – particularly subtype-2 (SSTR2) – are highly expressed in nearly all human meningiomas (7). I am editing the texting again as I did in my draft I sent back – please make these changes.

The objective of this systematic review was to study the prevalence of intracranial meningiomas in patients with GH-secreting pituitary adenomas or exposure to exogenous GH therapy. Furthermore, this review aimed to identify patient and tumor factors that predict the development of metachronous or synchronous intracranial meningiomas in patients with acromegaly.

## Materials and methods

This systematic review was registered on Prospero (ID CRD42022376998) and conducted with adherence to PRISMA statement guidelines.

### Eligibility criteria

Studies eligible for inclusion met the following criteria: 1) discussion of both GH (e.g., secreting tumors, replacement, treatment) and meningioma, 2) written in English or with available English translation, and 3) published in a peer-reviewed journal. There was no time limitation for included studies. Animal studies, studies published in non-English languages, and systematic reviews were excluded from the review.

### Search strategy

Searches were conducted using PubMed, EMBASE, and Web of Science. The following search terms were used: “growth hormone” OR “acromegaly” OR “growth hormone-secreting pituitary adenoma” OR “growth hormone secreting” OR “growth hormone producing” OR “growth hormone hypersecretion” OR “GH secreting” OR “GH producing” OR “GH hypersecretion” AND “meningioma” OR “meningiomas” OR “meningeal tumor” OR “meningeal tumors” OR “meningeal neoplasm” OR “meningeal neoplasms”.

### Data extraction

All abstracts identified in the initial literature search were screened for relevance by two independent investigators. The full text of selected articles was then reviewed to assess the eligibility of the study for inclusion in this systematic review. Data extraction was performed using Microsoft Excel. Information extracted included study location, study design, years including follow up, number (n) of individuals, % male, mean age. Patient factors extracted included genetics, family predisposition, comorbidities, radiation history, symptomatology, biochemical levels (e.g., GH, IGF-1), imaging modality and findings, surgical intervention, tumor remission/recurrence, and pathology. Data were synthesized and analyzed using linear regression analysis functions in Microsoft Excel and are presented in **Table 1** an elevated risk.

**Table 1:** Study information and patient factors for the 24 studies included in this systematic review.

Study	Radiation history	Other cancers	Order of onset	Age in y, mean ± SD (range)	Sex ratio M/F	Patients	Country	Preop GH (ng/mL)	Preop IGF-1 (ng/mL)	Meningioma size (cm^3^)	Meningioma location	Duration of GH exposure (y)
Thomas- Teinturier (8)	Yes	Non-meningioma brain tumors	Metachronous	4 (0–18)	1.58	40	France	NA	NA	NA	NA	6
Engelhardt (3)	Yes	33 (15%)	Synchronous	53	0.66	221	France	NA	NA	NA	Anterior convexity 3, posterior convexity 1, anterior/middle fossa 11, posterior fossa 1, falx cerebri 4	6
Gonzales- Virla (9)	Yes	NA	Metachronous	53 ± 13	0.29	94	Mexico	8.4	NA	0.904	NA	8
Zhao (10)	No	NA	Metachronous	58	0.00	2	China	15.9	21.2	0.67-13.3	Sella turcica	0
Burman (11)	Yes	Glioblastoma multiforme	Metachronous	40	1.16	253	31 countries	NA	NA	NA	Temporal convexity 4, frontal 3, parietal convexity 1, parasellar/sphenoidal 3, supratentorium 1, falx cerebri 1	0
Brignardello (12)	Yes	Papillary thyroid Ca, melanoma, basal cell Ca, spinal neurinoma	Metachronous	(0–10)	2.71	49	Italy	NA	NA	NA	NA	3-11
Patterson (13)	Yes	Glioma (6)	Metachronous	(0–15)	1.86	338	USA	NA	NA	NA	NA	NA
Ergun- Longmire (14)	Yes	Osteosarcoma, astrocytoma, glioma, mucoepidemoid Ca, adenocarcinoma, spindle cell sarcoma, papillary Ca	Metachronous	11 (1–21)	1.91	361	USA	14	NA	NA	NA	4
Jostel (15)	NA	Ependymoma	Metachronous	30 ± 13	NA	60	UK	NA	NA	1.3-3	NA	4.5
Drake (16)	No	NA	Metachronous	74	0	1	UK	10	489	4.81	NA	4.1
Chung (17)	Yes	NA	Synchronous	(30–75)	0.33	4	UK	NA	NA	NA	Temporal, extrasellar, intrasellar	4.6
De Menis (18)	No	NA	Metachronous	59	0	1	Italy	2.2	325	NA	NA	NA
Abs (19)	No	Goiter (3) and hemangioma (1)	Synchronous	(45–82)	0	7	Belgium	NA	NA	NA	Tuberculum sellae, frontal/occipital/parietal convexity, temporal fossa, sphenoid ridge parasellar, choroid plexus, sphenoid wing	NA
Bunick (20)	NA	NA	Synchronous	57	1.00	1	USA	28.6	NA	NA	Frontal	NA
Mathuriya (21)	No	NA	Synchronous	58	1	1	India	200	NA	NA	Frontal parasagittal	NA
Yarman (22)	No	NA	Synchronous	40	0	1	Turkey	90	80	NA	Falx	NA
Guaraldi (23)	No	NA	Metachronous	46	0	1	Italy	7.9	772	8.4	Parietal parasagittal	12
Herrero-Ruiz (24)	No	Pancreatic tumor; thyroid mass	Synchronous	35	0	1	Spain	84.1	702	2.4	Parietal	NA
Cannavo (25)	No	NA	Synchronous	47	0	1	Italy	82	807	NA	Right latero- and retrosellar	10
Curto (26)	No	NA	Metachronous	61	0	1	Italy	56	560	3.1	Right intracranial frontal	13
Kumaria (27)	No	NA	Synchronous	46	0	1	UK	NA	899	NA	Bilateral dural base	NA
Honegger (28)	NA	NA	Metachronous	49	0	1	Germany	170	NA	14.1	center parasagittal	5
Bolanowski (29)	NA	Large bronchial carcinoid	Metachronous	61	0	1	Poland	18.1	693	8	L cerebral hemisphere, supraventricular	10
Jawiarczyk-Prybytowska (30)	NA	Pulmonary adenocarcinoma, GIST, thyroid follicular adenoma, adrenal cortex adenoma, Renal carcinoma, multiple myeloma	Metachronous	68	0	1	Poland	NA	586	NA	Medullata oblongata tumor	NA

Ca, cancer; NA, not applicable; Preop, preoperative.

### NYU patients

Additional patients were included in a separate analysis using unpublished data from the investigators’ affiliated medical institution, NYU Langone Medical Center in Manhattan, New York. In conducting this analysis, we focused solely on patients with a diagnosis of both acromegaly and intracranial meningioma. Relevant cases were identified by searching the Department of Neurosurgery’s database of consecutive patients who had undergone resection of GH-secreting pituitary adenomas at NYU from December 2013 through March 2022. The electronic medical records (EMRs) of these patients were screened for imaging or chart evidence of meningioma. Additional cases were identified by searching through the database of patients with acromegaly currently or previously managed in NYU’s outpatient endocrinology clinics. The EMRs of these patients were screened for imaging or chart evidence of meningioma. We assessed 54 patients in the NYU system and identified 4 as having a diagnosis of both acromegaly and intracranial meningioma (**Table 2**). This portion of the study was approved by the IRB of NYU.

**Table 2:** Characteristics of four patients from NYU.

	Patient 1	Patient 2	Patient 3	Patient 4
**Sex**	M	F	F	F
**Age**	41	26	77	56
**Order of onset**	Synchronous	Synchronous	Metachronous	Metachronous
**Pituitary tumor pathology**	GH, cytokeratin CAM5.2, chromogranin, KI-67	GH, PIT-1, MIB1, CAM5.2	NA	GH, PIT-1, CAM5.2
**Meningioma pathology**	Anaplastic hemangiopericytoma, WHO grade III; +SSTR2, CD34, BCL-2, CD99; negative GH, CAM5.2	Surgery not performed	Surgery not performed	WHO grade I
**Meningioma location**	Convexity	Sellar/left parasellar	Right parasagittal superior sagittal sinus at anterior frontal lobe	Tuberculum sella
**Meningioma size (cm^3^)**	8.8	24	0.125	NA
**Preop IGF-1 (ng/mL)**	967	836	975	NA
**Preop GH (ng/mL)**	23.8	274	7.8	NA
**Family history**	No	Pituitary adenoma in maternal niece	No	Breast cancer and thyroid dx in mother
**Personal cancer history**	No	No	No	Breast cancer (BRCA1+)
**Radiation exposure**	No	No	No	No

dx, disease; NA, not applicable; Preop, preoperative, WHO, World Health Organization.

## Results

**Figure 1:**
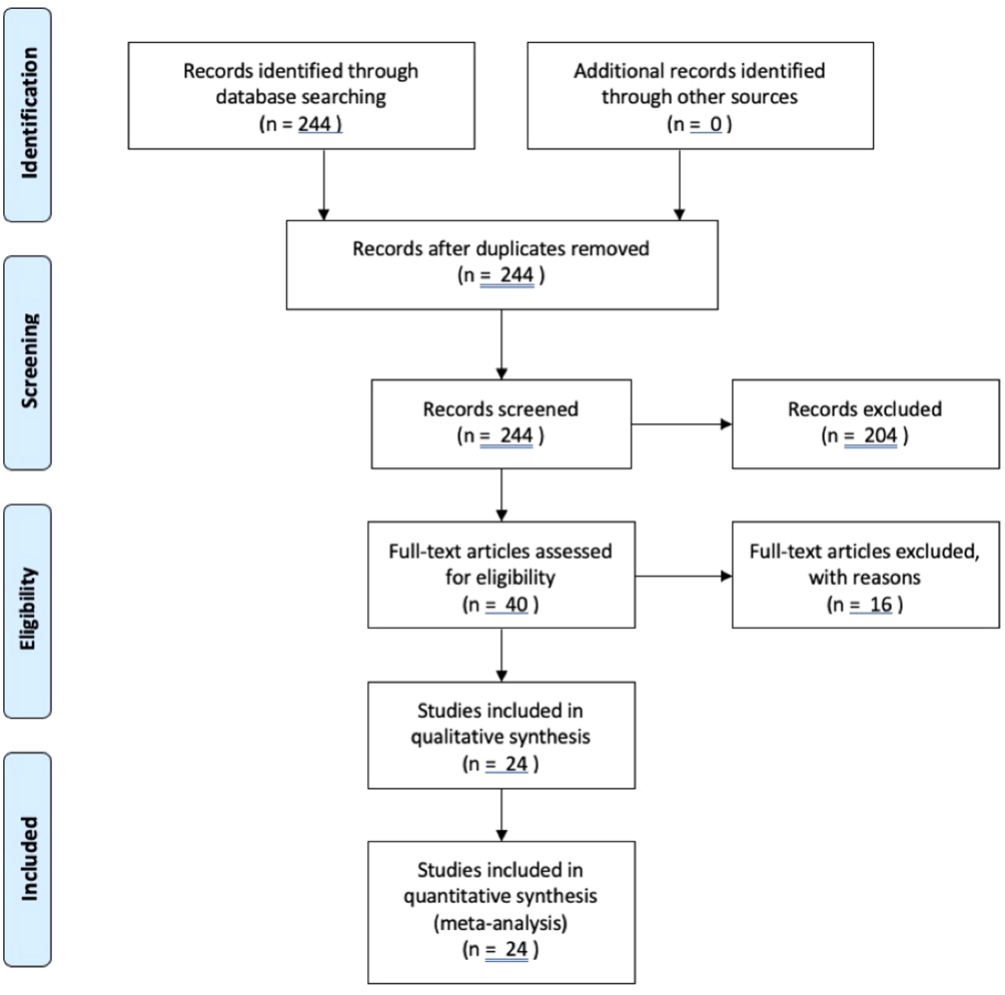
Systematic review flowchart.

**Figure 2:**
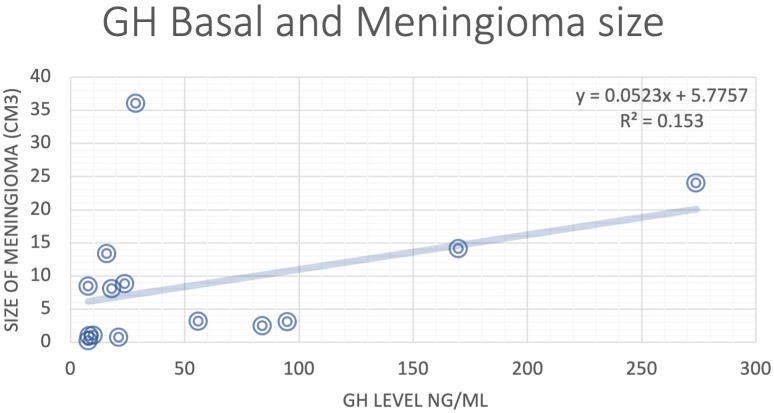
Correlation analysis for basal GH level and the size of the meningioma.

**Figure 3:**
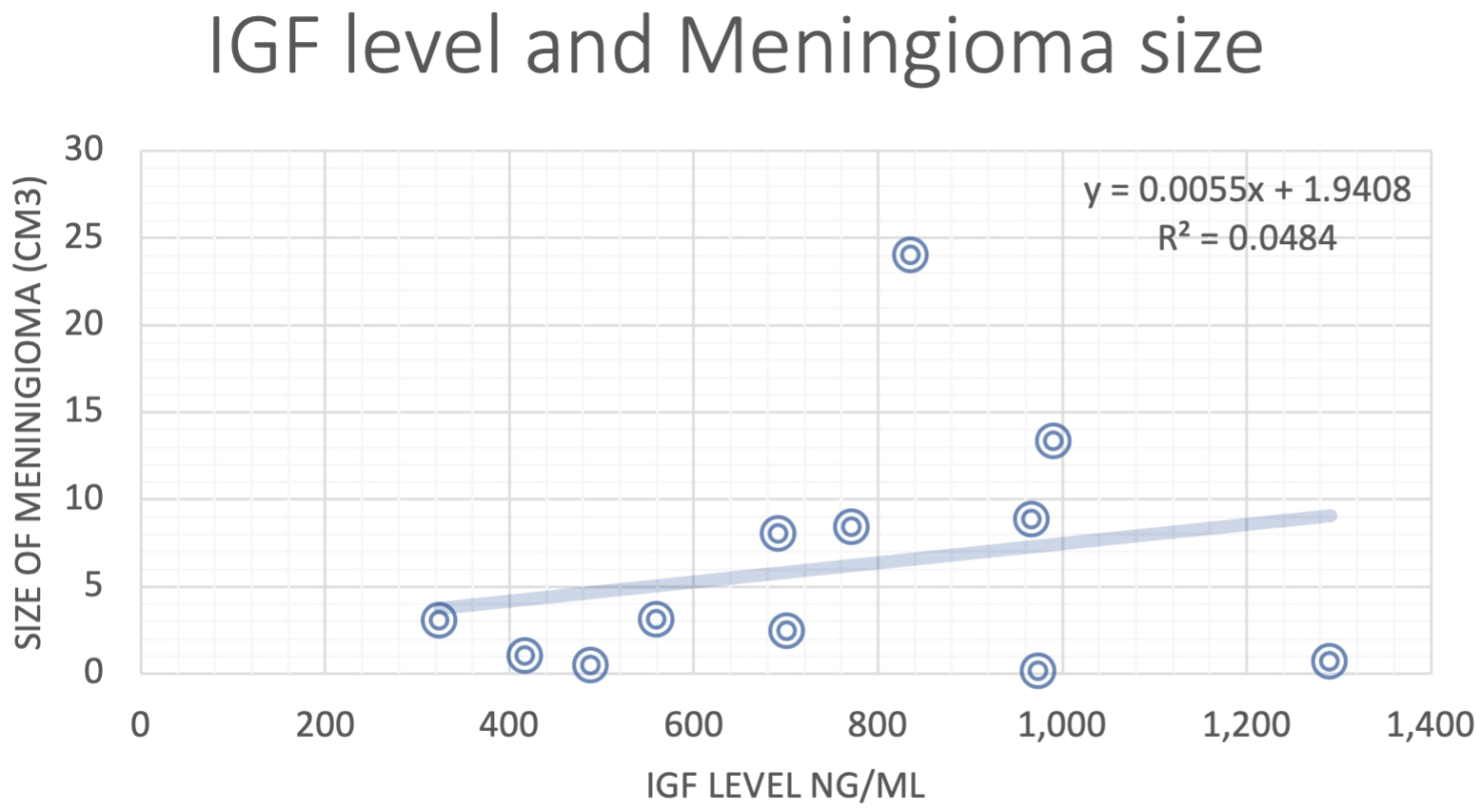
Correlation analysis for IGF level and the size of the meningioma.

Analysis of data from literature review showed that GH and IGF-1 levels did not have strong correlations with meningioma size (**Figure 1**). There was a weak positive correlation between IGF-1 level and GH basal level and meningioma size, but the sample size was relatively small even with integration of NYU patient data (**Figures 2**, **3**).

**Figure 4:**
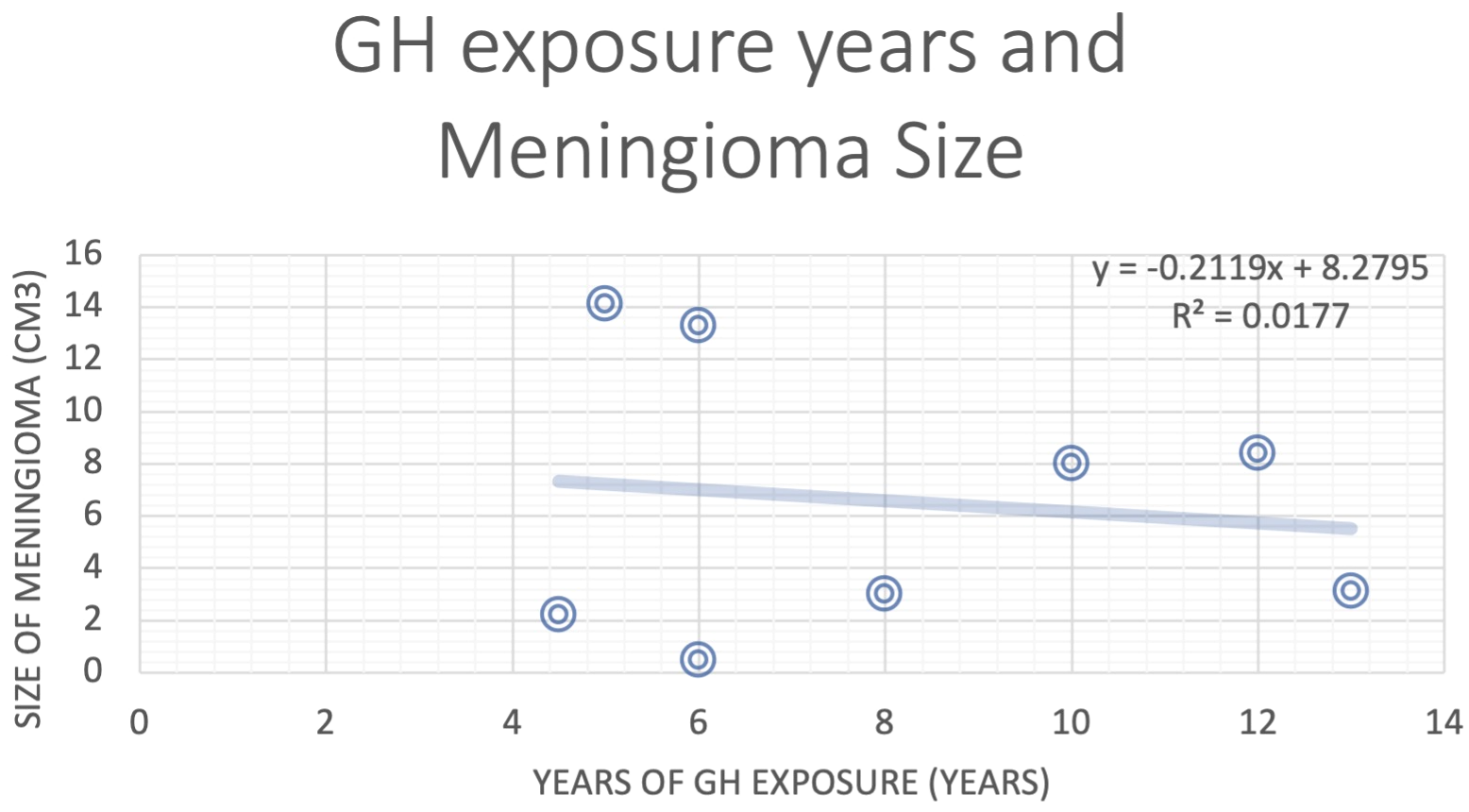
Correlation analysis for years of GH exposure and the size of the meningioma.

Roughly half of the patients included in the review developed meningioma after acromegaly (metachronous), and the other half were diagnosed with meningioma and acromegaly simultaneously (synchronous). (**Table 1**). For the metachronous patients in the studies that also reported their meningioma sizes and GH exposure years, we explored the correlation between the years elapsed between the two diagnoses and the size of the meningioma but discovered no clear correlation (**Figure 4**). However, this analysis was also limited by the small size of the study.

The locations of the meningiomas that co-occurred with acromegaly did not follow an obvious pattern (**Tables 1**, **2**). The location of the development of meningioma seemed to randomly occur from frontal to parietal lobe and from falx to sphenoid wing.

Data from NYU patients (**Table 2**) followed the same pattern observed in the literature; half of the patients had synchronous meningioma and acromegaly and half had metachronous presentation. The locations of meningiomas did not follow any specific pattern. Due to the scarcity of data, not many conclusions can be drawn regarding IGF-1 and GH levels and meningioma size.

## Discussion

Researching the incidence of meningioma in a rare cohort of acromegaly patients is a challenging task. This systematic review studies the prevalence of meningiomas in patients with GH excess. The review is limited by -patient case reports, making it difficult to identify a causal association. Assessment of possible causality is further limited by the use of radiation in a large cohort of aggressive GH-producing pituitary adenomas, which poses a confounding risk for the development of intracranial meningiomas. While previous case reports comment on a synchronous and metachronous development of meningiomas, the timing of onset is difficult to verify given the insidious disease course and delay in acromegaly diagnosis.

Surgery is the first line of treatment in patients with symptomatic or compressive meningiomas. However, tumors deemed surgically unresectable owing to location or extension into vital neurovascular structures may require radiotherapy (31). While there are currently no approved medications for treatment of meningiomas, the use of targeted therapy to help with recurrent or extensive meningiomas is certainly an attractive concept.

The strong expression of SSTR2 is well documented in intracranial meningiomas. Another pathway with a dominant role in meningioma development is that of Pi3K/Akt/mTOR (32, 33). Loss of function of the Merlin protein – a negative regular of mTOR encoded by the NF2 gene – leads to overactivation of this pathway and subsequent meningioma oncogenesis. Inactivation of the NF2 gene is one of the primary chromosomal changes observed in meningioma cells (34). Activation of SSTR2 can downregulate the Pi3K/Akt pathway. One agent with such action is octreotide, a somatostatin analog currently used to suppress tumor activity in acromegaly and other neuroendocrine tumors. Pasireotide is a newer agent with greater affinity for the SSTR5 receptor (35). In a study by Graillon el al.comparingthe effects of pasireotide to those of octreotide *in vitro* on meningioma primary cell cultures, combining an SST analog with an mTOR inhibitor like everolimus has demonstrated efficacy in treating aggressive meningiomas. These two agents together impose a strong synergistic inhibitory effect on oncogenic pathways in meningioma cells (36).

This phenomenon raises an important confounding variable in our study: exposure to somatostatin analogs in our study populations. Somatostatin Receptor Analogs (SRLs) are commonly used as adjuvant therapy in patients with acromegaly experiencing residual disease following neurosurgical resection of the adenoma. SRLs are even used as first-line therapy in certain cases of high surgical risk or low likelihood of surgical cure due to extrasellar tumor extension (37). The use of SRL, octreotide was not disclosed in any of the articles studied in the review, but it is highly likely that several of the patients in these studies were treated with octreotide, given how commonly this agent is used in acromegaly therapy. This possibility is important to note because SRLs can interfere with the natural history of meningiomas by downregulating the Pi3K/Akt pathway. One of the four NYU patients received a trial of octreotide for treatment of acromegaly approximately 24 years prior to meningioma diagnosis. However, we cannot draw meaningful conclusions regarding the effect of SRLs on meningioma development on the basis of a single patient.

Our review suggests that hereditary cancer syndromes might have a role in the co-occurrence of acromegaly and meningiomas. In nearly half of the articles studied, patients reported additional tumors (separate from pituitary adenoma and meningioma) including papillary thyroid cancer, osteosarcoma, hemangioma, skin tumors, and other CNS tumors such as glioblastoma, astrocytoma, and ependymoma. The presence of multiple tumor diagnoses raises the possibility that patients in this cohort were afflicted by familial cancer syndromes. A study by Engelhardt et al. showed that acromegaly patients with multiple endocrine neoplasia type 1 (MEN-1) were at significantly higher risk of developing a meningioma (11.7% vs 7%) compared to patients without this inherited disorder (3). A separate study by Asgharian demonstrated that meningiomas were 11 times more common in patients with Zollinger-Ellison syndrome (ZES) and MEN-1 than in patients with ZES alone (38). These findings suggest that hereditary cancer syndromes such as MEN-1 may have a potentiating effect on the relationship between meningiomas and GH-secreting pituitary adenomas.

In this literature review, no significant correlation was found between GH or IGF-1 level and meningioma size. GH and IGF-1 receptors are expressed in 88-94% of human meningiomas, across all histological grades (4). For this reason, IGF-1 upregulation is thought to be the primary mechanism underlying the co-occurrence of GH-secreting pituitary adenomas and intracranial meningiomas. However, the findings of this study raise the possibility that a different biologic mechanism might be at play. The various other receptors commonly expressed in meningioma cells – and their interplay with the metabolic consequences of acromegaly – should also be considered. These immunohistochemical markers include progesterone receptors, vascular endothelial growth factor, and caspase-3, which are detected in 87%, 69%, and 30% of meningiomas, respectively (4).

While no clear correlation between GH or IGF-1 level and meningioma size was identified in our review, previous studies have suggested that these hormones play a dominant role in the pathogenesis of meningiomas. Puduvalli et al. examined the *in vitro* action of fenretinide, a synthetic retinoid, against primary meningioma cultures and discovered that one mechanism by which this agent induces apoptosis in tumor cells is via inhibition of IGF-1-induced proliferation (39). In that study, meningioma cells were treated with IGF-1 alone, fenretinide alone, or a combination of both agents. A dose-dependent increase in cell proliferation was seen with IGF-1 compared to untreated controls. In the combination cultures, fenretinide suppressed the proliferative effect of IGF-1 on tumor cells. Thus, it is feasible that the correlation between IGF-1 level and meningioma size – a relationship well-established in prior literature – is dampened in our review by the effect of agents like retinoids and other undisclosed chemicals in our pooled patient population. This possibility highlights the importance of exploring the potential role of other pathophysiologic mechanisms.

Conflicting reports of the role of GH and IGF-1 receptors in meningioma oncogenesis are found in the literature. Blockage of these receptors (e.g., with the GH receptor antagonist pegvisomant) slows the growth of meningioma cells in both *in vitro* and *in vivo* mice studies (5, 6). This observation suggests that upregulation of IGF-1 due to GH hypersecretion in acromegaly may induce the appearance and growth of meningiomas. However, in one case report of a patient with acromegaly and meningioma (metachronously diagnosed one decade apart), volume of the meningioma increased over 10-fold during a 5-year period of treatment with pegvisomant (16).

Radiation-induced meningiomas are a very delayed adverse effect of brain irradiation. One study of 253 patients identified a mean latency period of 36 years between initial radiation exposure and meningioma diagnosis (40). The vast majority of patients were exposed in early childhood. Another study of 49 survivors of childhood leukemia treated with cranial irradiation found that 22% developed meningiomas as adults, with a mean latency period of 26 years (41). In the two metachronous NYU patients, one was diagnosed with the two tumors 8 years apart and the other was diagnosed 24 years apart. Neither patient had any reported history of radiation exposure. Acromegaly is typically diagnosed in the fifth decade of life, and meningioma is most often diagnosed in the sixth decade of life. Given the long latency period, it is unlikely that use of radiation to treat a pituitary tumor would lead to growth of meningioma a decade later. Thus, the effect of this confounding factor can be presumed to be minimal at most however larger studies are required to answer this question definitively.

Implications of a larger-scale replication study could be far-reaching, extending from screening guidelines to pharmacologic breakthroughs. Given that other studies have demonstrated higher incidence of intracranial meningiomas in patients with acromegaly compared to the general population, it is reasonable to adjust current clinical practice guidelines for acromegaly to include screening for meningioma at time of diagnosis and regular intervals thereafter (3). As magnetic resonance imaging of the brain is already a common part of the diagnostic workup for acromegaly, adding this recommendation to the guidelines should not add a significant cost burden. Still to be determined, however, is an appropriate frequency and duration (even after remission is achieved) of screening for meningioma in patients with acromegaly. Although this study was unable to ascertain the pathophysiologic etiology of the co-occurrence of these two tumors, further investigation into these mechanisms could revolutionize treatment for both conditions. A potential consideration is the use of SRLs and GH receptor antagonists for treatment of intracranial meningiomas based on future prospective studies.

## Limitations

An important limitation of our study pertains to radiation exposure Ionizing radiation is a strong risk factor for meningiomas and other CNS neoplasms (35). For patients with metachronous patterns of acromegaly and meningioma, interim exposure to cranial irradiation may confound the relationship between these two tumors. Though not the primary treatment modality, pituitary irradiation is often used in the management of GH-secreting pituitary adenomas, particularly in cases that are refractory to first-line surgical and pharmacologic interventions (42). Few of the studies in our review disclosed information regarding radiation exposure, making it difficult to ascertain how substantial a role this factor played in the findings we observed.

The main limitation of this study is the relatively small sample size of pooled patients in our systematic analysis, which is due in part to the overall rarity of co-occurrence of these two tumors in the general population. The correlation between the level of GH and size of meningioma is difficult to study in such a small sample. There is a lack of high-quality, large cohort studies examining how GH and GH inhibitors affect meningioma growth. Such studies could provide valuable data to study the biological relationship between these two conditions.

## Conclusion

Despite the limitations of the small sample size of the study there could be a positive correlation between GH and IGF-1 levels and meningiomas in patients with coexisting tumors. Future prospective studies in larger groups of patients can help delineate the mechanism of this correlation and helping with management of this rare condition.

## Data availability statement

The original contributions presented in the study are included in the article/supplementary material. Further inquiries can be directed to the corresponding author.

## Author contributions

AG: Data curation, Methodology, Writing – original draft, Writing – review & editing, Investigation, Formal analysis. AJ: Data curation, Formal analysis, Investigation, Methodology, Writing – original draft, Writing – review & editing. DP: Data curation, Validation, Resources, Project administration, Writing – review & editing. NA: Data curation, Investigation, Methodology, Writing – original draft, Writing – review & editing, Conceptualization, Project administration, Resources, Supervision, Validation.
